# Development of a 9-months pregnant hybrid phantom and its internal dosimetry for thyroid agents

**DOI:** 10.1093/jrr/rrt223

**Published:** 2014-02-09

**Authors:** E. Hoseinian-Azghadi, L. Rafat-Motavalli, H. Miri-Hakimabad

**Affiliations:** Physics Department, School of Sciences, Ferdowsi University of Mashhad, Azadi Square, Mashhad, 91775-1436, Iran

**Keywords:** internal dosimetry, thyroid agents, hybrid phantom, pregnancy, reference phantom

## Abstract

As a consequence of fetal radiosensitivity, the estimation of internal dose received by a fetus from radiopharmaceuticals applied to the mother is often important in nuclear medicine. A new 9-months pregnant phantom based on magnetic resonance (MR) images tied to the International Commission on Radiological Protection (ICRP) reference voxel phantom has been developed. Maternal and fetal organs were segmented from a set of pelvic MR images of a 9-months pregnant subject using 3D-DOCTOR^TM^ and then imported into the 3D modeling software package Rhinoceros^TM^ for combining with the adult female ICRP voxel phantom and further modeling. Next, the phantom organs were rescaled to match with reference masses described in ICRP Publications. The internal anatomy of previous pregnant phantom models had been limited to the fetal brain and skeleton only, but the fetus model developed in this study incorporates 20 different organs. The current reference phantom has been developed for application in comprehensive dosimetric study in nuclear medicine. The internal dosimetry calculations were performed for thyroid agents using the Monte Carlo transport method. Biokinetic data for these radiopharmaceuticals were used to estimate cumulated activity during pregnancy and maternal and fetal organ doses at seven different maximum thyroid uptake levels. Calculating the dose distribution was also presented in a sagittal view of the pregnant model utilizing the mesh tally function. The comparisons showed, in general, an overestimation of the absorbed dose to the fetus and an underestimation of the fetal thyroid dose in previous studies compared with the values based on the current hybrid phantom.

## INTRODUCTION

Protection of a developing fetus against ionizing radiation is of particular interest. Estimations of the absorbed radiation dose to a fetus from a nuclear medicine procedure performed on the mother are an important component of stochastic risk assessment. The pregnant patient or worker has a right to know the magnitude and type of potential radiation effects that might result from *in utero* exposure. If fetal doses are above 1 mGy, a more detailed explanation should be given [1].

Stylized versions of pregnant phantoms were previously developed by Stabin *et al.* [2], and subsequent organ refinements made by Chen [3], where fetal anatomy was modeled as an outer cylindrical shell of fetal skeleton with hemispherical ends, with an inner volume filled with generic fetal soft tissue. Although used widely for reporting doses in diagnostic nuclear medicine [4], this simplified treatment of fetal anatomy does not permit detailed assessment of the dose to many fetal skeletal and tissue structures [5]. On the other hand, in the 2007 Recommendations of the ICRP [6] it is suggested that doses from external and internal sources should be calculated using reference computational phantoms of the human body based on medical tomographic images, replacing the use of various mathematical models.

An image-based specimen-specific model of a 30-weeks pregnant woman, presented by Shi and Xu [7], was constructed from segmented computed tomography (CT) images. In 2008, Angel *et al.* published a 24-patient retrospective study in which voxelized models of female abdominal anatomy were created covering a range of gestational ages of 5–36 weeks [8]. These voxelized models were restricted to the region of the abdomenn and pelvis of a pregnant woman.

Then Xu *et al.* [9] published the first fully hybrid series of pregnant female models at the gestational ages of 12, 24 and 36 weeks. The fetal internal anatomy of these models was still limited to the fetal brain and skeletal model. No further organs of fetal anatomy had been incorporated into these pregnant female phantoms.

Recently, hybrid versions of human fetus were developed by Maynard *et al.* for the fetal ages of 8, 10, 15, 20, 25, 30, 35 and 38 weeks post-conception [5]. The Maynard *et al.* series of fetal hybrid computational phantoms contain fetal organs, bone-specific details at various ages and weight percentiles, however the fetus models were not located within a pregnant female model.

For reporting internal doses for image-based models of pregnant woman as Russell *et al.* [4] did for mathematical models, we decided to construct a series of hybrid computational phantoms of pregnant females that included fetal organs and real maternal internal anatomy according to magnetic resonance (MR) images. In this paper we present the first member of our series: a model of a 9-months pregnant phantom together with estimations of internal dosimetry for validating the phantom. The current reference version can provide internal dosimetric data applicable to nuclear medicine.

## MATERIALS AND METHODS

### Phantom construction

#### MR image sets

A set of MR images of a 9-months pregnant patient was obtained from Picture Archive Communication System (PACS) at Qaem Hospital, Mashhad, Iran. The pregnant subject was imaged using clinical MR. The soft tissue of the 38-weeks' fetus was sufficiently visualized under MR scan (performed on a Siemens Magnetom MRI system with 1.5 T static field strength), and an acceptable image quality for anatomical modeling was obtained. Independent sagittal, axial and coronal T2 weighted scans were performed with parameters as follows: image matrix of 256 × 256; number of 40, 40 and 35 planes for sagittal, axial and coronal scans, respectively; slice thickness of 6 mm; slice gap of 1.2 mm; pixel width of 1.5 mm for axial and sagittal scans; and 1.6 mm for coronal scan.

#### Segmentation of MR image sets

**Fig. 1.**
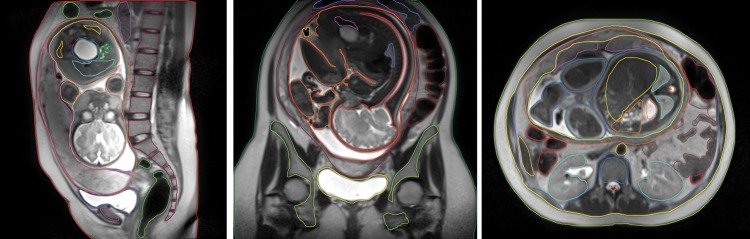
Segmentation of sagittal, coronal and axial MR image sets using 3D-Doctor software.

Segmentation of the MR image sets of the pregnant patient was performed using 3D-DOCTOR^TM^ (Able Software Corp., Lexington, MA), a 3D modeling and image-processing software package. The sagittal, axial and coronal images were imported into 3D-DOCTOR, and the anatomical structures of interest were contoured manually using a computer mouse (Fig. 1). Once all the necessary contours had been completed, the export boundary function was used to export the 3D contours as a Drawing Exchange file (*.dxf). 3D-DOCTOR's complex surface rendering functions also provided a polygon mesh model for each image set. Each polygon mesh model was exported as a Wavefront Object file, a format that is easily imported into most 3D modeling software packages. (For more details please see Appendix A.)

#### NURBS and polygon mesh modeling and merging to ICRP reference phantom

**Fig. 2.**
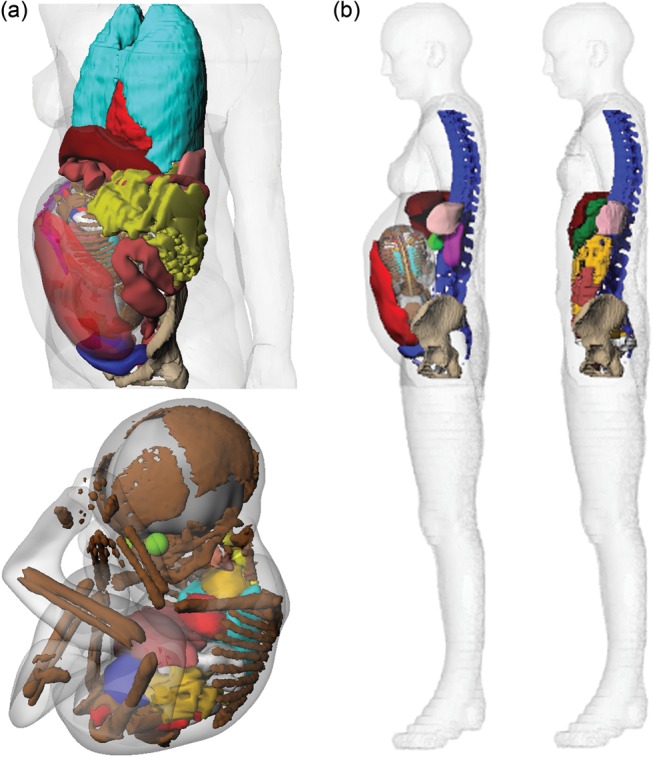
(**a**) NURBS and polygon mesh modeling of the pregnant female and her fetus combined with the female ICRP reference voxel phantom. (**b**) Female base phantom in comparison with current model.

The female base phantom used in development of the pregnant hybrid phantom was a BREP version of the ICRP reference voxel phantom. Following the completion of segmentation, the polygon mesh ‘Wavefront Object’ files of the ICRP female phantom and MR image sets were imported into ‘Rhinoceros^TM^’ (McNeel North America, Seattle, WA), a NURBS and polygon mesh modeling software package. Organs of the abdominal and pelvic region of the base phantom were replaced with those of the MR image sets. The polygon mesh models of maternal small intestine, spine, pelvis and fetal internal organs were left unaltered, while other organs were converted to NURBS surfaces. These surfaces are a powerful modeling tool that allows precise deformation of individual volumes, which is a useful feature, particularly in regards to computational phantom construction [5]. In constructing the pregnant phantom, ‘Rhinoceros’ was used to (i) correctly orient the polygon mesh models, and (ii) incorporate NURBS surfaces into the phantom for various modeling requirements, including repair of segmentation artifacts, imparting deformability to certain structures, and adding skeletal bones and soft tissues that were not initially segmented. NURBS and polygon mesh modeling of the pregnant phantom are presented in Fig. 2a and b. Replaced NURBS and polygon mesh models of maternal and fetal organs were then volumetrically rescaled to target volumes based on ICRP Publications 110 and 89. Careful manipulations of NURBS control points permit minimizing of the overlapped structures within the current version. (Further details of the NURBS and polygon mesh modeling methodology are provided in Appendix B.)

#### Voxelization

**Fig. 3.**
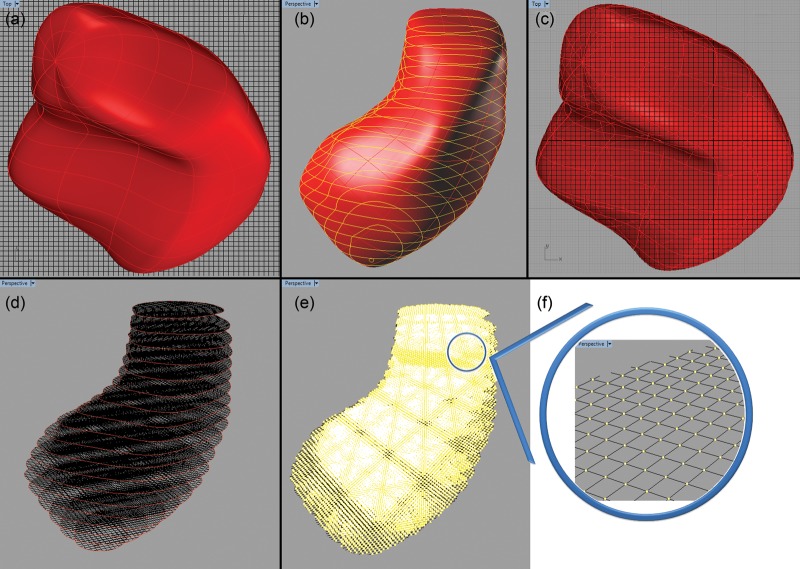
(**a**) Drawing network of lines intersecting at the center of voxels and visual inspection of Object (stomach) for voxelization process. (**b**) Contouring the object by using the Rhinoceros ‘contour’ tool. (**c**, **d**) Selecting the contours as a cutting object in the trimming process and trimming the lines. (**e**) Intersecting the remaining lines and exporting points.

The voxelization process of the hybrid pregnant phantom was done using an innovative method developed by our research group. Rhinoceros simple functions were used in this method to voxelize each organ. A network of lines was drawn with their intersections located in the center of the voxels. Figure 3a shows the object to be voxelized later from the top viewport. The Rhinoceros ‘contour’ tool was used to extract the contours of the object (Fig. 3b). All the created contours were perpendicular to the *z*-axis, and the distance between them was equal to the voxel *z*-dimension. The Rhinoceros ‘trim’ tool was then applied to delete the lines' network outside the contours (Fig. 3c and d). So, the remaining lines of the network intersect at the center of the voxels that are located inside the object. The points could be easily extracted using the Rhinoceros ‘intersect’ tool (Fig. 3e) and then exported as a text file.

This voxelization method is efficient because one only has to draw the network of lines once. Another advantage of this method is visual inspection and manipulation of voxels during the voxelization process. Some tissues such as skin, walls of stomach, small intestine, large intestine, gall bladder, urinary bladder and the cortical region of the pelvis and spine were added to the phantom after voxelization by using a FORTRAN program. For example, four layers of voxels at the surface of stomach were assigned as stomach wall. It is important to note that volumes obtained after the rescaling process were calculated directly from Rhinoceros^TM^; these values may not be identical to the volumes calculated after voxelization. Thus, a FORTRAN program was applied to adjust reference volumes after voxelization by adding or removing a few voxels to the exterior layers of organs.

### Calculation of cumulated activities

The cumulated activities from two references were used in this study to estimate organ doses for the current model [4, 10]. Russell *et al.* [4] reported these values as residence times of radiotracer in source organs during pregnancy, and considered the fetus and/or the placenta in source regions. But, the biokinetic data published in ICRP 53 [10] are assumed to represent the average normal adult, and thus the non-pregnant woman. Generally, the assumption was made that no changes occurred in the biodistribution of the radiopharmaceutical in maternal organs during pregnancy [4]. So, in order to evaluate organ doses, cumulated activities assigned to the fetus and/or placenta were applied from Russell *et al.*'s publication.

In addition, Russell *et al.* [11] provided a biokinetic model of iodine during pregnancy. This compartmental model was used to estimate cumulated activities of ^131^iodine and ^123^iodine by the method introduced in MIRD pamphlet No. 12 [12]. A maple program was developed to solve the differential equations of the compartmental model. The cumulated activities of urinary bladder contents and the kidneys were obtained by using the method published in ICRP 53 [10].

### Estimation of organ doses and Monte Carlo calculations

In this study, the organ doses were calculated from the Monte Carlo simulations, which were carried out separately for photons and electrons. A general-purpose Monte Carlo code, MCNPX version 2.4.0, was employed to calculate the absorbed dose for the pregnant model [13]. The phantom was incorporated into the MCNPX lattice file. Organ- and tissue-specific densities and elemental compositions were implemented into the material card of the MCNPX code.

The source regions were defined separately in each run: thyroid, stomach (stomach wall and contents), small intestine (small intestine wall and contents), kidneys, liver, bladder contents, salivary glands, maternal remaining tissues, fetal thyroid and remaining tissues. Gastrointestinal contents (apart from stomach and small intestine contents) were not included in source regions. Next, the total organ doses resulting from photons and electrons emitted from ten source regions were obtained.

The simulations provided the dose (MeV/g), i.e. energy deposition (MeV) per unit mass (g), in each target organ (*T*) per emitted particle. The dose per particle was multiplied by the total photon or electron yield per decay and summed to obtain the absorbed dose (mGy/MBq). The dose was scored using the track length estimate of the heating tally (F6) for photons (kerma approximation) and energy deposition (+F6) for electrons. Photons and electrons (mode P E) were transported when the emitted particle was an electron, while only photons were transported (mode P) for photons emitted from the source region.

The spectra published in ENSDF decay data [14] with yields >0.1% was employed for the estimation of the organ doses. The beta spectrum of ^131^I was approximated using the Fermi function with respect to the maximum energy of beta. The auger electrons were determined by their average energy, and the conversion electrons by their maximum energy in subshells. Absorbed doses to active red marrow and the endosteal region (bone surface) were estimated by using the F6 tally and the masses of active marrow in bone sites, as published in ICRP Publication 116 [15]. For a more detailed investigation, additional MCNPX mesh tallies were used to graphically display the dose in voxels of the model in sagittal view. Mesh tally type 1 with the ‘pedep’ option and type 3 with the ‘total’ option were used for photon and electron sources, respectively, which score the average energy deposition per unit volume (MeV/cm^3^/source-particle). The visualization was performed in two arrays of 159 × 348 crossing at the *i* = 149 and *i* = 176 of the phantom. A rectangular grid exactly overlaid on the lattice geometry was defined. Therefore, the energy deposited per unit volume in each voxel could be converted to the energy per unit mass by dividing by each voxel's density. The energy deposited per unit mass (MeV/g/source-particle) in each voxel was then converted to the absorbed dose (mGy/MBq) for visualization of the dose distribution map.

## RESULTS

### Organs masses vs reference data

The organ masses after the voxelization process of the adult female ICRP reference phantom (AF) [16] and the pregnant model developed in this project were compared in Table 1. The total mass of the model, which was calculated from multiplying the voxel volume by its density, was 68.1 kg while for the AF phantom it was 60.0 kg. Considering the fact that much effort was taken to adjust replaced organ masses to the reference values obtained during 3D modeling, the differences are obviously low in most cases. Certain organs, such as the uterus, breasts, skin and adipose tissue will have increased masses during pregnancy, as indicated in this table, while the gastrointestinal contents have decreased in volume. The discrepancies in other organs are <1%, except in the gall bladder wall and the trunk muscles, which have discrepancies of <3%. ICRP Publication 89 reference masses were carefully adopted for fetal organs, and the differences are <0.3% (see Table 1). The total mass of the fetus is 3.471 kg, which differs from the reference value of 3.5 kg by ∼0.8%. The mass of the uteroplacental unit and its contents is 4.8 kg.

**Table 1.** Organ masses of adult female ICRP Reference voxel phantom in comparison with the current model and their differences

Organs of interest	ICRP 110 Reference mass (g)	New mass (g)	Difference (%)	Density comment	Material comment
Cartilage, trunk	313.61	313.61	0.00	^1^	^1^
Breast, left, adipose tissue	150.00	489.73	226.48	^1^	^1^
Breast, right, adipose tissue	150.00	512.11	241.40	^1^	^1^
Gall bladder wall	10.24	9.96	−2.74	^1^	^1^
Gall bladder contents	45.75	31.82	−30.45	^1^	^1^
Stomach wall	140.00	140.00	0.00	^1^	^1^
Stomach contents	230.01	165.84	−27.90	^1^	^1^
Small intestine wall	600.00	600.00	0.00	^1^	^1^
Small intestine contents	280.01	156.61	−44.07	^1^	^1^
Ascending colon wall	90.00	90.00	0.00	^1^	^1^
Ascending colon contents	100.01	86.42	−13.59	^1^	^1^
Transverse colon wall, right	55.00	55.00	0.01	^1^	^1^
Transverse colon contents, right	60.00	58.06	−3.23	^1^	^1^
Transverse colon wall, left	55.00	54.79	−0.37	^1^	^1^
Transverse colon contents, left	30.00	28.51	−4.96	^1^	^1^
Descending colon wall	90.00	90.00	0.00	^1^	^1^
Descending colon contents	50.00	49.00	−2.03	^1^	^1^
Sigmoid colon wall	45.01	45.01	−0.01	^1^	^1^
Sigmoid colon contents	80.00	79.01	−1.23	^1^	^1^
Rectum wall	25.00	24.85	−0.56	^1^	^1^
Kidney, left, pelvis	149.48	149.48	0.00	^1^	^1^
Kidney, right, pelvis	125.53	125.53	0.00	^1^	^1^
Liver	1 400.00	1 399.72	−0.02	^1^	^1^
Lung, left, tissue	377.02	377.02	0.00	^1^	^1^
Lung, right, tissue	472.03	471.85	−0.04	^1^	^1^
Muscle, trunk	8 518.22	8 389.34	−1.51	^1^	^1^
Esophagus (wall)	34.99	34.99	0.01	^1^	^1^
Ovary, left	5.50	5.50	0.03	^1^	^1^
Ovary, right	5.50	5.50	0.03	^1^	^1^
Pancreas	120.00	120.00	0.00	^1^	^1^
Residual tissue, trunk	11 803.12	14 149.25	19.88	^1^	^1^
Skin, trunk	1 004.12	1 114.05	10.95	^1^	^1^
Spleen	130.00	130.00	0.00	^1^	^1^
Urinary bladder wall	40.00	40.00	−0.01	^1^	^1^
Thyroid	17.00	17.00	0.00		
Urinary bladder contents	200.00	226.53	13.26	^1^	^1^
Uterus	79.99	823.33	929.30	^1^	^1^

Organs of interest	ICRP 89 Reference mass (g)	New mass (g)	Difference (%)	Density comment	Material comment
Amniotic fluid		207.17		^2^*	^7^
Umbilical cord		25.60		^1^*	^1^*
Placenta		1 070.39		^3^	^6^
Fetus	3 500	3 471.98	0.80		
Fetus, brain	370	370.00	0.00	^2^, ^5^	^5^
Fetus, skeleton		204.31		^2^	^4^
Fetus, eyes		4.13		^2^	^1^
Fetus, spinal cord		5.43		^2^	^1^
Fetus, thyroid	1.3	1.30	0.24	^2^	^1^
Fetus, lungs	60	59.87	0.21	^2^	^1^
Fetus, thymus	13	13.00	−0.03	^2^	^1^
Fetus, heart	20	20.00	0.01	^2^	^1^
Fetus, liver	130	130.30	−0.23	^2^	^1^
Fetus, kidneys	25	25.00	−0.02	^2^	^1^
Fetus, adrenals	6	6.00	0.00	^2^	^1^
Fetus, spleen	9.5	9.50	0.00	^2^	^1^
Fetus, SI wall		13.63		^2^	^1^
Fetus, SI contents		11.39		^2^	^1^
Fetus, LI wall		6.03		^2^	^1^
Fetus, LI contents		5.95		^2^	^1^
Fetus, bladder wall		9.44		^2^	^1^
Fetus, bladder contents		27.22		^2^	^1^
Fetus, stomach wall		10.15		^2^	^1^
Fetus, stomach contents		3.86		^2^	^1^
Fetus, gall bladder wall		0.72		^2^	^1^
Fetus, gall bladder contents		0.72		^2^	^1^
Fetus, pancreas	5	4.99	0.11	^2^	^1^
Fetus, soft tissue		2 299.96		^2^	^3^
Fetus, skin		229.08		^1^	^1^

^1^ICRP 110: adult female densities and materials of given organ or tissue. The last two columns are comments regarding tissue compositions and mass densities. ^1^*ICRP 110: density and material of blood. ^2^Maynard *et al.*: 38 weeks densities and materials of given organ or tissue. ^2^*Maynard *et al.*: density of gastrointestinal contents. ^3^Xu *et al.*: density and material of fetus soft tissue. ^4^Xu *et al.*: density and material of skeleton. ^5^Xu *et al.*: density and material of brain. ^6^Xu *et al.*: density and material of placenta. ^7^Xu *et al.*: density and material of uterine contents.

The soft tissue densities and material compositions of the current pregnant phantom were primarily adapted from ICRP Publication 110 [16], which provides these data for the adult female ICRP reference phantom. In the case of fetal tissues, suitable surrogates were adopted. The ‘Density and Material comment’ columns provide a numerical key to data references (e.g. fetal soft tissue density was taken from Maynard *et al.*, while material composition was taken from Xu *et al.*) (see Table 1 and its footnotes for further details).

### Cumulated activities for pregnant female model

The calculation of radiation dose estimates to the fetus is important for radiation protection purposes. To obtain the best estimates of the radiation dose to the fetus, the best biological and physical models should be employed. In this paper, the best available biokinetic model [4, 11] for a pregnant woman was used to calculate cumulated activities for maternal thyroid, salivary glands, stomach, small intestine, liver, bladder contents, kidneys, feces and remaining tissues, in addition to fetal thyroid and the remaining fetal organs. The biokinetic model was employed assuming a 5, 15, 25, 35, 45, 55 and 95% maximum thyroid uptake level. Tables 2 and 3 show the cumulated activities calculated in this investigation in comparison with those published by two other references [4, 10]. ICRP Publication 53 [10] provides cumulated activities for normal patients at six different maximum thyroid uptakes within the range of 5–55%. A 95% thyroid uptake was added in this study to include hyperthyroid patients. This model applies to the intravenous administration of iodide; however, the oral administration of iodide will delay the appearance of iodide in the blood by 10–15 min, which will have only a minimal effect on the activity level in the blood and little effect on the thyroid uptake [10]. This compartmental biokinetic model includes fetal thyroid and fetal organic compartments. The model applied in this study, describes the behavior of administered iodide and organically bound iodine (in T3 and T4 form), which is released to the body tissues from the thyroid, while dose estimates reported by ICRP Publication 53 [10] do not consider the effects of organically bound iodine. In this paper, in addition to other source organs, the activities cumulated in salivary glands and in the fetal thyroid were assessed separately, whereas previous publications [4, 10] do not account for these vital organs in dose estimates. This comparison is presented in Tables 2 and 3.

**Table 2.** Cumulated activities for ^131^I sodium iodide obtained in this study to estimate internal doses in comparison with two other references

^131^I	This study							Russell *et al.*	ICRP 53
	5%	15%	25%	35%	45%	55%	95%	25%	25%
Thyroid	12.27 h	1.55 d	2.61 d	3.73 d	4.64 d	5.76 d	10.41 d	2.54 d	2.53 d
Salivary glands	32.20 min	29.04 min	25.84 min	22.42 min	19.67 min	16.27 min	2.14 min		
Stomach	1.61 h	1.45 h	1.29 h	1.12 h	59.00 min	48.82 min	6.43 min	1.33 h	1.66 h
SI	1.82 h	1.65 h	1.46 h	1.27 h	1.11 h	55.33 min	7.29 min	1.5 h	1.66 h
Liver	51.37 min	1.16 h	1.46 h	1.79 h	2.05 h	2.37 h	3.71 h	1.16 h	
Bladder contents	1.67 h	1.50 h	1.33 h	1.15 h	1.01 h	50.64 min	24.74 min	1.92 h	1.32 h
Kidney	4.80 min	4.80 min	4.80 min	4.80 min	4.80 min	4.80 min	4.80 min		5.7 min
Feces	<30 s	<30 s	<30 s	<30 s	<30 s	<30 s	<30 s		
Remaining tissues	6.36 h	6.33 h	6.30 h	6.27 h	6.25 h	6.21 h	6.08 h	6.4 h	7.72 h
Fetus–thyroid	5.50 h	4.96 h	4.42 h	3.83 h	3.36 h	2.78 h	21.99 min	4.72 h	
Remaining–Fetus	23.09 min	20.82 min	18.53 min	16.07 min	14.11 min	11.67 min	1.54 min

**Table 3.** Cumulated activities for ^123^I sodium iodide obtained in this study to estimate internal doses in comparison with two other references

^123^I	This study							Russell *et al.*	ICRP 53
	5%	15%	25%	35%	45%	55%	95%	25%	25%
Thyroid	37.94 min	1.97 h	3.41 h	5.04 h	6.43 h	8.25 h	17.37 h	3.28 h	2.94 h
Salivary glands	21.00 min	19.49 min	17.84 min	15.98 min	14.39 min	12.31 min	1.89 min		
Stomach	1.05 h	58.46 min	53.53 min	47.94 min	43.17 min	36.93 min	5.66 min	54.42 min	1.08 h
SI	1.19 h	1.10 h	1.01 h	54.33 min	48.92 min	41.85 min	6.42 min	1.03 h	1.08 h
Liver	26.57 min	24.82 min	22.94 min	20.81 min	19.00 min	16.66 min	4.72 min	6.78 min	
Bladder contents	1.06 h	58.98 min	54.35 min	49.08 min	44.58 min	38.58 min	8.46 min	1.25 h	50 min
Kidney	3.39 min	3.39 min	3.39 min	3.39 min	3.39 min	3.39 min	3.39 min		3.7 min
Feces	<0.01 s	<0.01 s	<0.01 s	<0.01 s	<0.01 s	<0.01 s	<0.01 s		
Remaining tissues	3.98 h	3.69 h	3.39 h	3.04 h	2.75 h	2.36 h	25.53	2.92 h	5.03 h
Fetus–thyroid	19.04 min	17.65 min	16.16 min	14.47 min	13.03 min	11.15 min	1.71 min	16.26 min	
Remaining–fetus	19.80 s	18.40 s	16.85 s	15.08 s	13.57 s	11.62 s	1.78 s		

The biokinetic model was not provided for Tc-99m pertechnetate in Russell *et al.* [4, 11] or in ICRP Publication 53 [10], so the cumulated activities were not computed in this paper separately but taken from those references (Table 4).

**Table 4.** Cumulated activities for Tc-99m sodium pertechnetate from two references used in this study to estimate internal doses

Tc-99m		Russell *et al.*	ICRP 53
Thyroid		2.28 min	2.23 min
Salivary glands			3.35 min
Stomach wall		18.18 min	14.9 min
Stomach contents			9.24 min
SI contents			25.3 min
Right colon = ULI	wall	47.40 min	32.6 min
	contents		44.6 min
Left colon = LLI	wall	29.40 min	
	contents		21.8 min
Kidneys			2.00 min
Bladder contents		47.70 min	20.7 min
Remaining tissues		5.02 h	4.32 h
Fetus		20.04 min	
Placenta		57.24 min	

### Internal dose estimates

Organ doses from ^131^I and ^123^I distributed within a 9-month pregnant patient were calculated at seven levels [5, 15, 25, 35, 45, 55 and (extremely) 95%] of thyroid uptake and presented in Tables 5 and 6. In addition, organ doses were obtained for the present model, by using cumulated activities from two other references [4, 10]. In the last column of the Tables 5 and 6, the reported dose to the 9-month fetus and fetal thyroid for the stylized model were also tabulated [4, 17].

**Table 5.** Organ doses (mGy/MBq) calculated for this model with different sets of cumulated activities, in addition to fetal doses reported by previous publications

^131^I Sodium Iodide	This study							^a^	^b^
Thyroid uptake	5%	15%	25%	35%	45%	55%	95%	25%	25%
Active red marrow	7.55E-02	1.48E-01	2.22E-01	3.01E-01	3.64E-01	4.42E-01	7.68E-01	2.17E-01	2.17E-01
Colon	6.38E-02	6.46E-02	6.55E-02	6.64E-02	6.71E-02	6.81E-02	7.25E-02	6.51E-02	6.59E-02
Lungs	9.35E-02	1.86E-01	2.79E-01	3.79E-01	4.60E-01	5.59E-01	9.72E-01	2.73E-01	2.77E-01
Stomach wall	7.12E-01	6.55E-01	5.97E-01	5.36E-01	4.86E-01	4.25E-01	1.70E-01	6.14E-01	7.48E-01
Breasts	4.69E-02	8.63E-02	1.26E-01	1.69E-01	2.03E-01	2.46E-01	4.22E-01	1.24E-01	1.25E-01
Ovaries	6.46E-02	6.04E-02	5.63E-02	5.20E-02	4.85E-02	4.44E-02	3.29E-02	6.99E-02	6.14E-02
Urinary bladder wall	1.52E-01	1.39E-01	1.26E-01	1.12E-01	1.01E-01	8.77E-02	5.25E-02	1.72E-01	1.32E-01
Esophagus	4.30E-01	1.23E + 00	2.05E + 00	2.92E + 00	3.62E + 00	4.49E + 00	8.09E + 00	2.00E + 00	1.99E + 00
Liver	1.20E-01	1.64E-01	2.09E-01	2.57E-01	2.96E-01	3.44E-01	5.43E-01	1.81E-01	7.95E-02
Thyroid	8.17E + 01	2.48E + 02	4.17E + 02	5.97E + 02	7.42E + 02	9.20E + 02	1.66E + 03	4.06E + 02	4.04E + 02
Endosteal region	4.36E-02	7.63E-02	1.10E-01	1.45E-01	1.73E-01	2.09E-01	3.55E-01	1.07E-01	1.09E-01
Salivary gland	9.72E-01	1.04E + 00	1.10E + 00	1.17E + 00	1.23E + 00	1.30E + 00	1.58E + 00	3.77E-01	3.76E-01
Adrenal	6.94E-02	7.89E-02	8.86E-02	9.90E-02	1.07E-01	1.18E-01	1.61E-01	8.68E-02	8.17E-02
Kidney	9.59E-02	9.86E-02	1.01E-01	1.04E-01	1.07E-01	1.10E-01	1.22E-01	6.45E-02	1.10E-01
Pancreas	9.61E-02	9.77E-02	9.93E-02	1.01E-01	1.02E-01	1.04E-01	1.11E-01	1.02E-01	1.09E-01
SI-wall	3.48E-01	3.18E-01	2.88E-01	2.57E-01	2.31E-01	1.99E-01	6.82E-02	2.92E-01	3.21E-01
Spleen	8.04E-02	8.67E-02	9.31E-02	9.99E-02	1.05E-01	1.12E-01	1.40E-01	9.21E-02	1.02E-01
Thymus	3.62E-01	1.05E + 00	1.74E + 00	2.49E + 00	3.08E + 00	3.82E + 00	6.89E + 00	1.70E + 00	1.70E + 00
Uterus	7.66E-02	7.32E-02	6.98E-02	6.62E-02	6.34E-02	5.98E-02	4.65E-02	7.27E-02	7.34E-02
Remainder tissues	1.32E-01	2.41E-01	3.51E-01	4.69E-01	5.63E-01	6.80E-01	1.17E + 00	3.41E-01	3.47E-01

^**131**^**I Sodium Iodide**	This study							^a^	^b^	^c^
Thyroid uptake	5%	15%	25%	35%	45%	55%	95%	25%	25%	25%
Placenta	5.79E-02	5.50E-02	5.22E-02	4.91E-02	4.67E-02	4.37E-02	3.31E-02	6.58E-02	6.53E-02	
Fetus	3.08E-01	2.81E-01	2.55E-01	2.26E-01	2.03E-01	1.75E-01	5.85E-02	2.41E-01	2.40E-01	2.70E-01
Fetus, brain	1.11E-01	1.02E-01	9.28E-02	8.28E-02	7.47E-02	6.48E-02	2.49E-02	2.43E-01	2.41E-01	
Fetus, skeleton	1.35E-01	1.27E-01	1.19E-01	1.10E-01	1.03E-01	9.45E-02	5.88E-02	2.32E-01	2.32E-01	
Fetus, eyes	1.08E-01	9.95E-02	9.12E-02	8.23E-02	7.51E-02	6.63E-02	3.01E-02	2.48E-01	2.48E-01	
Fetus, spinal cord	3.65E-01	3.32E-01	2.99E-01	2.64E-01	2.36E-01	2.01E-01	5.68E-02	2.46E-01	2.48E-01	
Fetus, thyroid	4.35E + 02	3.92E + 02	3.49E + 02	3.03E + 02	2.65E + 02	2.20E + 02	2.90E + 01	1.12E-01	1.13E-01	2.70E + 02
Fetus, Lungs	2.07E-01	1.91E-01	1.74E-01	1.56E-01	1.41E-01	1.23E-01	4.92E-02	3.50E-01	3.52E-01	
Fetus, thymus	1.25E + 00	1.13E + 00	1.01E + 00	8.79E-01	7.75E-01	6.46E-01	1.12E-01	2.50E-01	2.51E-01	
Fetus, heart	2.18E-01	2.01E-01	1.83E-01	1.64E-01	1.49E-01	1.30E-01	5.16E-02	2.59E-01	2.60E-01	
Fetus, Liver	9.32E-02	8.83E-02	8.33E-02	7.80E-02	7.38E-02	6.85E-02	4.68E-02	2.52E-01	2.53E-01	
Fetus, kidneys	7.67E-02	7.56E-02	7.44E-02	7.32E-02	7.22E-02	7.10E-02	6.61E-02	2.55E-01	2.56E-01	
Fetus, adrenals	9.37E-02	8.96E-02	8.55E-02	8.11E-02	7.75E-02	7.31E-02	5.49E-02	2.58E-01	2.60E-01	
Fetus, spleen	1.08E-01	1.03E-01	9.87E-02	9.39E-02	9.00E-02	8.52E-02	6.54E-02	2.66E-01	2.70E-01	
Fetus, SI wall	8.51E-02	8.41E-02	8.31E-02	8.20E-02	8.12E-02	8.01E-02	7.59E-02	2.63E-01	2.63E-01	
Fetus, LI wall	6.58E-02	6.67E-02	6.77E-02	6.88E-02	6.96E-02	7.06E-02	7.51E-02	2.54E-01	2.54E-01	
Fetus, bladder wall	6.46E-02	6.48E-02	6.51E-02	6.53E-02	6.55E-02	6.58E-02	6.70E-02	2.55E-01	2.54E-01	
Fetus, stomach wall	1.15E-01	1.09E-01	1.03E-01	9.68E-02	9.17E-02	8.53E-02	5.90E-02	2.63E-01	2.65E-01	
Fetus, gall bladder wall	7.52E-02	7.23E-02	6.94E-02	6.63E-02	6.39E-02	6.08E-02	4.82E-02	2.52E-01	2.52E-01	
Fetus, pancreas	9.86E-02	9.51E-02	9.16E-02	8.78E-02	8.48E-02	8.10E-02	6.55E-02	2.65E-01	2.67E-01	
Fetus, soft tissue	1.56E-01	1.45E-01	1.33E-01	1.21E-01	1.11E-01	9.92E-02	4.93E-02	2.40E-01	2.39E-01	
Fetus, skin	7.77E-02	7.41E-02	7.04E-02	6.65E-02	6.34E-02	5.95E-02	4.41E-02	2.07E-01	2.07E-01	

^a,b^Organ dose estimates using cumulated activities from Russell *et al.* and ICRP Publication 53, respectively. ^c^Dose to the fetus and fetal thyroid reported by Russell *et al.* [4] and [17], respectively.

**Table 6.** Organ doses (mGy/MBq) calculated for this model with different sets of cumulated activities, in addition to fetal doses reported by previous publications

^123^I Sodium Iodide	This study							^a^	^b^
Thyroid uptake	5%	15%	25%	35%	45%	55%	95%	25%	25%
Active red marrow	8.87E-03	1.05E-02	1.22E-02	1.42E-02	1.59E-02	1.81E-02	2.91E-02	1.14E-02	1.25E-02
Colon	1.19E-02	1.11E-02	1.03E-02	9.34E-03	8.54E-03	7.50E-03	2.25E-03	9.70E-03	1.15E-02
Lungs	9.73E-03	1.16E-02	1.35E-02	1.58E-02	1.77E-02	2.02E-02	3.26E-02	1.20E-02	1.46E-02
Stomach wall	1.02E-01	9.49E-02	8.72E-02	7.84E-02	7.09E-02	6.11E-02	1.21E-02	8.75E-02	1.04E-01
Breasts	5.20E-03	5.66E-03	6.15E-03	6.72E-03	7.20E-03	7.83E-03	1.10E-02	5.34E-03	6.83E-03
Ovaries	1.81E-02	1.68E-02	1.55E-02	1.40E-02	1.27E-02	1.10E-02	2.31E-03	1.93E-02	1.64E-02
Urinary bladder wall	3.81E-02	3.55E-02	3.27E-02	2.95E-02	2.68E-02	2.32E-02	5.02E-03	4.34E-02	3.19E-02
Esophagus	1.94E-02	4.40E-02	7.05E-02	1.00E-01	1.26E-01	1.59E-01	3.27E-01	6.67E-02	6.26E-02
Liver	1.76E-02	1.67E-02	1.58E-02	1.48E-02	1.39E-02	1.27E-02	6.85E-03	8.61E-03	9.47E-03
Thyroid	7.47E-01	2.32E + 00	4.01E + 00	5.93E + 00	7.56E + 00	9.70E + 00	2.04E + 01	3.86E + 00	3.46E + 00
Endosteal region	5.85E-03	6.43E-03	7.05E-03	7.75E-03	8.35E-03	9.14E-03	1.31E-02	6.43E-03	7.68E-03
Salivary gland	1.08E-01	1.04E-01	1.00E-01	9.50E-02	9.08E-02	8.53E-02	5.76E-02	1.03E-02	1.02E-02
Adrenal	1.53E-02	1.44E-02	1.34E-02	1.24E-02	1.15E-02	1.03E-02	4.29E-03	1.08E-02	1.40E-02
Kidney	1.74E-02	1.66E-02	1.58E-02	1.49E-02	1.41E-02	1.30E-02	7.84E-03	9.47E-03	1.79E-02
Pancreas	2.37E-02	2.22E-02	2.05E-02	1.86E-02	1.70E-02	1.49E-02	4.46E-03	1.91E-02	2.37E-02
SI-wall	4.93E-02	4.58E-02	4.20E-02	3.77E-02	3.41E-02	2.93E-02	5.36E-03	4.22E-02	4.57E-02
Spleen	2.19E-02	2.05E-02	1.91E-02	1.75E-02	1.61E-02	1.43E-02	5.16E-03	1.84E-02	2.28E-02
Thymus	1.50E-02	3.62E-02	5.91E-02	8.49E-02	1.07E-01	1.36E-01	2.80E-01	5.61E-02	5.26E-02
Uterus	1.09E-02	1.02E-02	9.35E-03	8.45E-03	7.68E-03	6.67E-03	1.61E-03	9.67E-03	1.06E-02
Remainder tissues	1.62E-02	1.84E-02	2.07E-02	2.33E-02	2.56E-02	2.85E-02	4.33E-02	1.85E-02	2.07E-02

^123^I Sodium Iodide	This study							^a^	^b^	^c^
Thyroid uptake	5%	15%	25%	35%	45%	55%	95%	25%	25%	25%
Amniotic fluid	9.06E-03	8.45E-03	7.81E-03	7.07E-03	6.44E-03	5.62E-03	1.48E-03	8.53E-03	9.20E-03	
Umblical cord	6.24E-03	5.87E-03	5.47E-03	5.02E-03	4.63E-03	4.13E-03	1.59E-03	5.92E-03	7.49E-03	
Placenta	8.81E-03	8.21E-03	7.56E-03	6.82E-03	6.19E-03	5.36E-03	1.20E-03	8.69E-03	8.94E-03	
Fetus	1.15E-02	1.07E-02	9.88E-03	8.93E-03	8.12E-03	7.07E-03	1.77E-03	9.24E-03	1.00E-02	9.80E-03
Fetus, brain	7.74E-03	7.20E-03	6.63E-03	5.97E-03	5.42E-03	4.68E-03	1.02E-03	8.67E-03	8.75E-03	
Fetus, skeleton	2.06E-02	1.92E-02	1.77E-02	1.61E-02	1.46E-02	1.28E-02	3.40E-03	1.81E-02	2.00E-02	
Fetus, eyes	6.31E-03	5.89E-03	5.43E-03	4.90E-03	4.46E-03	3.87E-03	9.45E-04	7.40E-03	7.97E-03	
Fetus, spinal cord	1.87E-02	1.74E-02	1.60E-02	1.44E-02	1.30E-02	1.13E-02	2.28E-03	9.54E-03	1.06E-02	
Fetus, thyroid	4.12E + 00	3.82E + 00	3.50E + 00	3.14E + 00	2.82E + 00	2.42E + 00	3.71E-01	7.04E-03	7.80E-03	2.90E + 00
Fetus, lungs	1.38E-02	1.28E-02	1.18E-02	1.07E-02	9.66E-03	8.37E-03	1.90E-03	1.15E-02	1.27E-02	
Fetus, thymus	4.10E-02	3.80E-02	3.49E-02	3.13E-02	2.82E-02	2.42E-02	4.20E-03	8.99E-03	9.92E-03	
Fetus, heart	1.28E-02	1.19E-02	1.10E-02	9.91E-03	9.00E-03	7.81E-03	1.85E-03	9.61E-03	1.07E-02	
Fetus, liver	7.01E-03	6.56E-03	6.07E-03	5.52E-03	5.05E-03	4.44E-03	1.37E-03	8.15E-03	9.04E-03	
Fetus, kidneys	1.02E-02	9.54E-03	8.85E-03	8.06E-03	7.40E-03	6.52E-03	2.14E-03	1.11E-02	1.26E-02	
Fetus, adrenals	1.18E-02	1.11E-02	1.02E-02	9.25E-03	8.43E-03	7.36E-03	2.01E-03	1.23E-02	1.38E-02	
Fetus, spleen	1.58E-02	1.47E-02	1.36E-02	1.23E-02	1.12E-02	9.80E-03	2.63E-03	1.55E-02	1.77E-02	
Fetus, SI wall	1.08E-02	1.01E-02	9.38E-03	8.56E-03	7.87E-03	6.96E-03	2.40E-03	1.14E-02	1.30E-02	
Fetus, LI wall	7.54E-03	7.09E-03	6.62E-03	6.08E-03	5.62E-03	5.02E-03	2.00E-03	8.94E-03	1.01E-02	
Fetus, bladder wall	6.23E-03	5.87E-03	5.48E-03	5.05E-03	4.68E-03	4.19E-03	1.75E-03	8.11E-03	9.05E-03	
Fetus, stomach wall	1.17E-02	1.09E-02	1.01E-02	9.15E-03	8.36E-03	7.32E-03	2.11E-03	1.17E-02	1.33E-02	
Fetus, gall bladder wall	5.96E-03	5.59E-03	5.19E-03	4.74E-03	4.35E-03	3.85E-03	1.32E-03	7.73E-03	8.56E-03	
Fetus, pancreas	1.26E-02	1.18E-02	1.09E-02	9.87E-03	9.02E-03	7.91E-03	2.34E-03	1.29E-02	1.47E-02	
Fetus, soft tissue	9.39E-03	8.76E-03	8.09E-03	7.33E-03	6.68E-03	5.83E-03	1.56E-03	8.53E-03	9.26E-03	
Fetus, skin	8.40E-03	7.85E-03	7.25E-03	6.58E-03	6.00E-03	5.25E-03	1.46E-03	8.75E-03	9.65E-03	

^a, b^Organ dose estimates using cumulated activities from Russell *et al.* and ICRP Publication 53, respectively. ^c^Dose to the fetus and fetal thyroid reported by Russell *et al.* [4] and [17], respectively.

Not all maternal organ doses were addressed here; only doses of critical maternal organs and fetal organs were considered in this research. As expected, the thyroid gland receives the largest maternal organ dose for ^131^I and ^123^I. The fetal radiation dose for this pregnant model at 25% uptake was estimated at 2.55 × 10^−1^ and 2.40 × 10^−1^ mGy/MBq, by using the cumulated activities of this study and Russell *et al.*, respectively, while this value was reported as 2.70 × 10^−1^ mGy/MBq for the stylized model (Table 5). This demonstrates that the published fetal dose has been overestimated in the mathematical model.

Fetal radiation doses were quantified for 20 different organs separately. As an example, the fetal thyroid dose was 349 mGy/MBq when assuming a maternal thyroid uptake of 25%, in comparison with the figure of 270 mGy/MBq reported for the mathematical version [4] (see Table 5). These comparisons reveal an underestimation of ∼25% of the absorbed dose to the fetal thyroid as reported for the stylized phantom compared with the values based on the current hybrid phantom.

It should be noted that organ doses have considerable variations due to different thyroid uptake. This issue has been addressed earlier for the adult female ICRP voxel phantom [18]. Of particular importance is the fetal thyroid dose, which decreases by a factor of 15 as a result of increased maternal thyroid uptake.

**Fig. 4.**
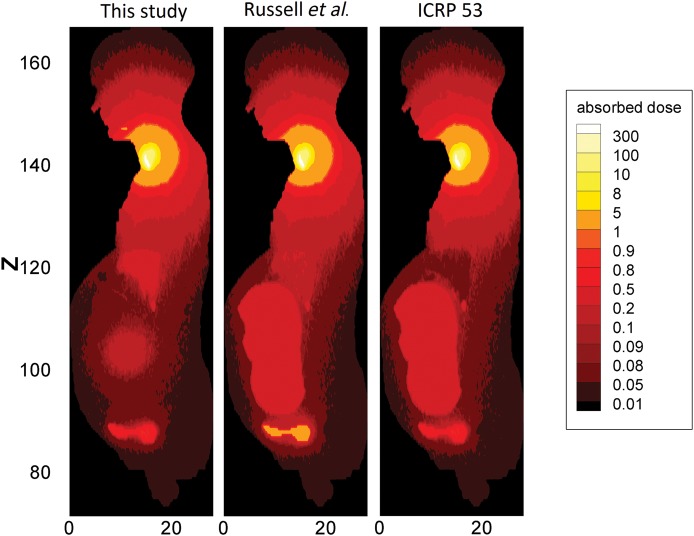
Comparison of dose maps calculated with three different sets of cumulated activities at 25% thyroid uptake. Both photons and electrons have been included in the calculations.

**Fig. 5.**
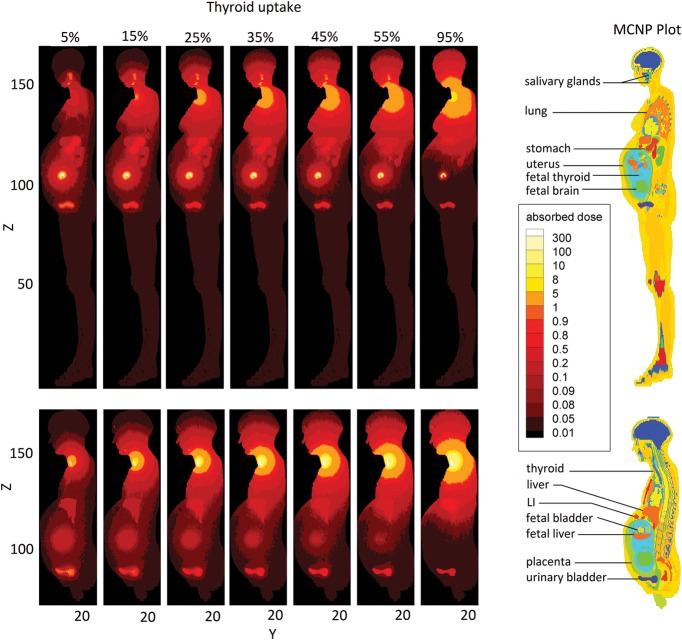
Comparison of dose maps of two sagittal planes of the phantom calculated at different maternal thyroid uptakes, including both photons and electrons contributions of ^131^I. Upper plots include fetal thyroid voxels and lower plots contain maternal thyroid voxels.

**Fig. 6.**
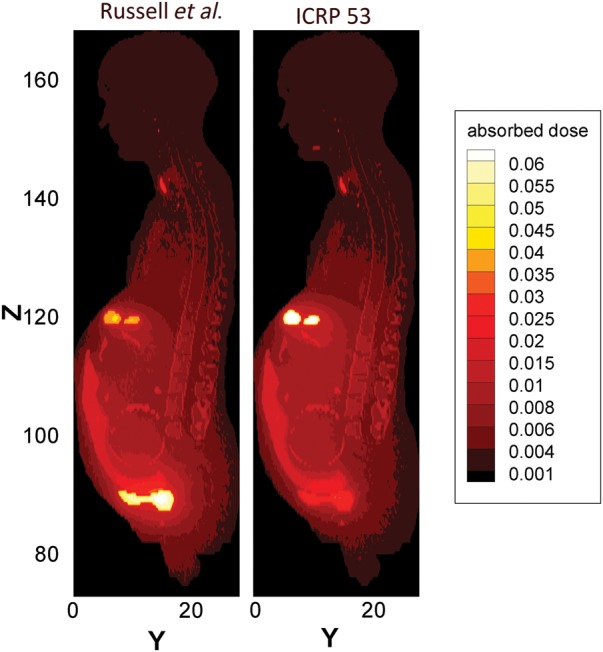
Comparison of dose maps calculated with two sets of cumulated activities involving both photons and electron contributions of Tc-99m.

To better clarify dose variations due to different cumulated activities, distributions of the dose in the pregnant woman and her fetus were computed and plotted in Figs 4–6. This 2D mesh tally provided the dose distribution map of two sagittal planes of the phantom, including either maternal or fetal thyroid voxels (Fig. 6). The positions and sizes of mesh tallies were carefully selected so that only one material was included in each mesh tally. Then, absorbed dose per unit activity administered to the mother was calculated for all the voxels, and the dose distribution was plotted. Figures 4–6 indicates a comparison between the dose distributions calculated with different biokinetic data. The comparison of dose distributions for three different sets of cumulated activities (this study, Russell *et al.* and ICRP Publication 53) at 25% thyroid uptake are shown in Fig. 4. In Fig. 5, a comparison between different thyroid uptakes has been made for ^131^I incorporation.

For the case of Tc-99-m pertechnetate, organ doses have been tabulated in Table 7. The first and second columns indicate the absorbed dose calculated for the current pregnant model by using cumulated activities from Russell *et al.* and ICRP Publication 53. The third column illustrates the fetal dose reported by Russell *et al*. The fetal dose was estimated at 9.31 × 10^−3^ mGy/MBq for this pregnant model, which was in good agreement with that of the stylized model [4]. The dose distributions are plotted in Fig. 6 for both sets of cumulated activities.

**Table 7.** Organ doses (mGy/MBq) calculated for this model with different sets of cumulated activities, in addition to fetal doses reported by previous publications

99m-Tc Sodium Pertechnetate	^a^	^b^		^a^	^b^	^c^
Active red marrow	5.28E-03	4.88E-03	Placenta	1.93E-02	1.85E-02	
Colon	2.95E-02	2.76E-02	Fetus	8.81E-03	9.48E-03	9.30E-03
Lungs	5.16E-03	4.90E-03	Fetus, brain	8.92E-03	8.34E-03	
Stomach wall	1.92E-02	2.93E-02	Fetus, skeleton	1.56E-02	1.70E-02	
Breasts	3.13E-03	3.11E-03	Fetus, eyes	8.22E-03	8.45E-03	
Ovaries	1.04E-02	6.75E-03	Fetus, spinal cord	7.52E-03	8.38E-03	
Urinary bladder wall	1.88E-02	1.10E-02	Fetus, thyroid	6.65E-03	6.99E-03	
Esophagus	4.31E-03	4.29E-03	Fetus, lungs	8.29E-03	9.34E-03	
Liver	4.97E-03	6.36E-03	Fetus, thymus	7.48E-03	8.10E-03	
Thyroid	2.63E-02	2.57E-02	Fetus, heart	7.69E-03	8.61E-03	
Endosteal region	3.69E-03	3.29E-03	Fetus, liver	7.61E-03	8.51E-03	
Salivary gland	1.44E-03	1.04E-02	Fetus, kidneys	7.78E-03	9.46E-03	
Adrenal	6.26E-03	7.51E-03	Fetus, adrenals	7.58E-03	9.19E-03	
Kidney	7.01E-03	1.02E-02	Fetus, spleen	8.26E-03	1.02E-02	
Pancreas	7.93E-03	9.81E-03	Fetus, SI wall	8.04E-03	9.65E-03	
SI-wall	7.05E-03	1.03E-02	Fetus, LI wall	7.79E-03	9.47E-03	
Spleen	6.96E-03	7.85E-03	Fetus, bladder wall	7.83E-03	9.28E-03	
Thymus	3.63E-03	3.36E-03	Fetus, stomach wall	7.76E-03	9.18E-03	
Uterus	8.22E-03	8.31E-03	Fetus, gall bladder wall	7.37E-03	8.35E-03	
Remainder tissues	5.69E-03	5.89E-03	Fetus, pancreas	7.97E-03	9.63E-03	
Amniotic fluid	8.71E-03	8.70E-03	Fetus, soft tissue	8.36E-03	9.08E-03	
Umbilical cord	8.19E-03	9.66E-03	Fetus, skin	8.73E-03	9.51E-03	

^a, b^Organ dose estimates using cumulated activities from Russell *et al.* and ICRP Publication 53, respectively. ^c^Dose to the fetus reported by Russell *et al.* [4].

## DISCUSSION

The current pregnant model is the first phantom that represents the whole body of a pregnant female and about 20 of the fetal organs. In addition, variations in the shape and volume of the soft tissues in the abdominopelvic cavity have been modeled as realistically as possible, according to MR images. The boundary representation method was used to form the current phantom. It demonstrates the ability of the hybrid construction process to model maternal and fetal anatomy from medical image sets with sufficient image quality. Construction of this model without using the hybrid approach would have been impossible due to the following requirements: (i) replacing the abdominal and pelvic region of the female ICRP reference voxel phantom with that of the pregnant model and matching the edges of overlapping organs; (ii) modeling the fetal skeletal bones and thyroid, which were poorly or not recognizable in the original MR image sets, in their correct anatomical location; (iii) imparting deformability to certain structures such as the liver, stomach, spleen, pancreas and gall bladder.

Biokinetic data during pregnancy were needed to calculate organ doses. Activities cumulated in the fetus and in the maternal source organs were published by Russell *et al.*, but they did not mention the fetal thyroid as a source organ because the stylized version of the pregnant phantom does not contain this organ. With regard to dose calculation, this tiny organ of 1.3 g mass is very important since fetal thyroid concentrates the iodine, which crosses the placenta. The fetal cumulated activity (4.72 h) reported by Russell *et al.* is in good agreement with the sum of those activities given in this study for both fetal thyroid and the remaining fetal organs (4.42 h + 0.31 h). It should be noted that 94% of cumulated activity assigned to the fetus belongs to the fetal thyroid. Assuming a uniformly distributed source in the fetus body, rather than a local source in the fetal thyroid, caused dose underestimation of ∼7%. The fetal dose was 2.55 × 10^−1^ mGy/MBq when the fetal thyroid was defined as a separate source compared with 2.40 × 10^−1^ mGy/MBq when the total fetus body considered as a uniform source. This occurs because more radiation would escaped from the fetus body when it was defined as a uniform source organ. More significant disagreements of about three orders of magnitude were observed for the fetal thyroid dose due to self-irradiation (see Tables 5 and 6). The absorbed dose to adjacent organs to the fetal thyroid, such as the brain, appears to increase by a factor of 3 when the source is defined locally in the thyroid. This issue is clearly indicated in the dose map plotted in Fig. 4.

Variations due to different uptakes are shown in Fig. 5. In general, organ doses close to the urinary bladder, and particularly fetus doses, tend to decrease throughout the uptake range. This occurs because the increase in maternal thyroid uptake causes radioiodine cumulated in the other organs to decline. The 95% uptake of maternal thyroid extreme particularly demonstrates this fact. This level of uptake was considered since fetal dose has not been well established for patients whose iodine kinetics differ from the standard model. Conversely, as thyroid uptake varies from 0% to 95%, the thyroid gland takes up more radioiodine; hence, the absorbed dose to the thyroid and its adjacent organs increases.

Discrepancies between organ doses with similar thyroid uptake should assign to different biokinetic data utilized in the calculations. For a thyroid uptake of 25%, a comparison was made between organ doses assessed using three different sets of cumulated activities. For example, the thyroid doses in ^131^I sodium iodide incorporation are 4.17 × 10^2^, 4.06 × 10^2^ and 4.04 × 10^2^ mGy/MBq calculated using three sets of cumulated activities (this study, Russell *et al.* and ICRP 53, respectively). On the other hand, the values of cumulated activity assigned to the thyroid are 2.61, 2.54 and 2.53 d, respectively (see Table 2). It is clear that there is a relationship between organ doses and cumulated activities. Indeed, the absorbed dose to source organs is larger when the cumulated activity assigned to the organ is higher.

For organs other than source regions, the nearest source region contributes mostly in organ dose, and its cumulated activity should be considered for the interpretation of results. As an example, absorbed doses to the ovaries for ^123^I incorporation calculated using three sets of cumulated activities are 1.55 × 10^−2^, 1.93 × 10^−2^ and 1.64 × 10^−2^ mGy/MBq, respectively. The values for the urinary bladder content (which is in the vicinity of the target organ) are 54.35 min, 1.25 h and 50 min, separately (see Table 3). It was expected that the lowest dose absorbed would be obtained using the third set (ICRP Publication 53), but according to the result (1.55 × 10^−2^) the minimum value was obtained using the first set of cumulated activities. This is due the fact that in the third set the fetus was defined as a uniform source organ, and as the distance between the source (fetus) and the target (ovary) thus decreased, absorbed dose to the ovaries increased.

In the case of Tc-99-m pertechnetate, good agreement was achieved for fetal dose between our calculations and those of Russell *et al.* [4], although the fetal organ doses are newly derived in this study. The dose distributions plotted in Fig. 6 reveal a ‘hot’ area in the placental region. This can be explained by the fact that the cumulated activity assigned to the placenta was greater than normalized cumulated activity of the remaining tissues. Fetal bones are distinguishable in this plot, which means that the absorbed dose to the fetal bones is higher than that of the fetal soft tissues as a result of their greater density.

## FUNDING

This study was supported by Vice President for Research and Technology of Ferdowsi University of Mashhad. (Grant no. 20425, 1/3/2012).

## Figures and Tables

**Fig. A1.**
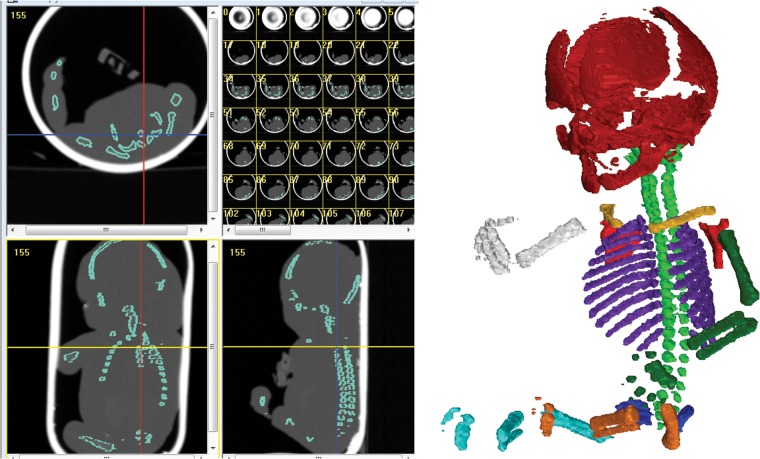
Segmentation and polygon mesh model of fetal skeleton.

**Fig. B1.**
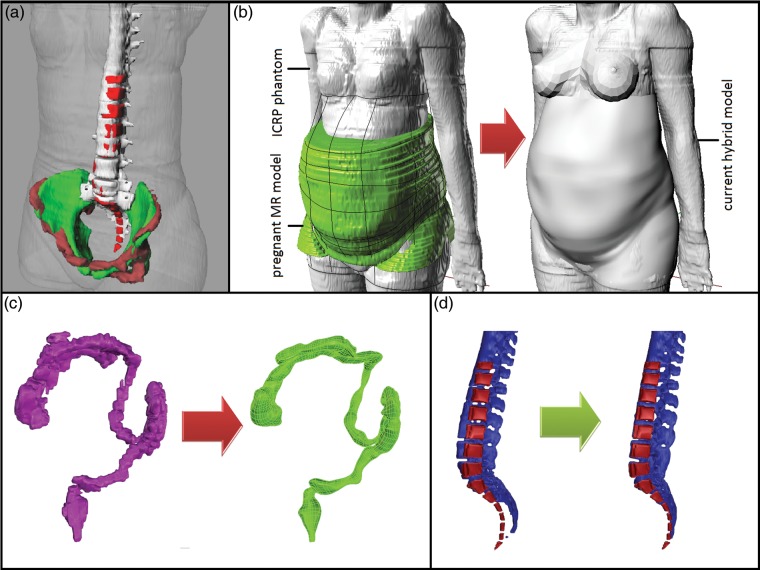
(**a**) Orientation of segmented structures (red) to the polygon mesh version of the adult female ICRP reference voxel phantom (green and gray) guided by the location of bones. (**b**, **c**) Creation of NURBS surface using ‘curve network’ tool. (**d**) Before (left) and after (right) orienting and aligning AF spine (blue) to the segmented spine landmark (red).

## APPENDICES

### Appendix A: Segmentation of MR image sets

Contours segmented from MR images included the maternal outline, exterior boundary of uterus, interior boundary of uterus, exterior boundary of muscles, interior boundary of muscles, cervix, urinary bladder, large intestine (LI), small intestine (SI), blood vessels, pelvis, spine, spinal cord, kidneys, spleen, liver, gall bladder, placenta and umbilical cord. Moreover fetal outline, brain, liver, heart, lungs, stomach, SI, LI, urinary bladder, kidneys, gall bladder, eyes, spinal cord, mandible and pelvis were segmented from MR images. Image contrast was limited in the fetal abdominal region. Consequently, fetal adrenals, spleen, pancreas, thymus and thyroid were later added manually during NURBS and polygon mesh modeling. Walls of the stomach, SI, LI, gall bladder and urinary bladder were later added during the voxelization process. The fetal skeleton was segmented from a previous CT scan of an aborted 5-month fetus and used in this model (Fig. A1).

## Appendix B: NURBS and polygon mesh modeling and merging to ICRP reference phantom

### Orientation

Ideally, the segmentation process would occur within a single image set, permitting correct orientation of the anatomy immediately upon being imported into another program. Because segmentation included three image sets (axial, coronal and sagittal), it was necessary to correctly orient and align the three sets of polygon objects. Those structures were then oriented to the polygon mesh version of the adult female ICRP reference voxel phantom (AF), a process guided by the skin contour and the locations of other bones such as pelvis and spine (Fig. B1).

### Organ replacement and NURBS surface construction

All organs of the abdominal and pelvic region of the base phantom were replaced with polygon mesh objects of the pregnant model (except for the pelvis, spine, adrenals and ovaries). Then, the following polygon mesh objects were converted to NURBS surfaces: maternal body outline, exterior boundary of uterus, interior boundary of uterus, exterior boundary of muscles, interior boundary of muscles, cervix, urinary bladder, urinary bladder contents, ureters, liver, stomach wall, stomach contents, gall bladder wall, pancreas, spleen, kidneys, LI, gall bladder and placenta, in addition to the fetal brain, eyes and spinal cord. As an example, creating of the maternal body outline and LI NURBS surfaces are illustrated in Fig. B1.

Conversion to NURBS surfaces occurred through one of two processes, depending on the geometry of the organ or structure. In the first process, segmented contours exported directly from 3D-Doctor as Drawing Exchange Files (*.dxf) were imported into Rhinoceros. These contours were used later as a curve network to construct NURBS surfaces. In the second process, Rhinoceros' ‘polyline on mesh’ tool was used to generate a network of curves around the polygon mesh object. Subsequent curve refinements should be performed using ‘rebuild’ and ‘curve edit’ tools to obtain smooth and optimum curves. The ‘curve network’ tool was then used to create a NURBS surface around the frame provided by that tool. In all cases, after conversion, the original polygon mesh objects were deleted from the phantom.

### Fetus

The fetal eyes were replaced by two NURBS spheres located exactly in their correct position. To account for the fetal adrenals, spleen, pancreas, thymus and thyroid gland in the phantom, previously developed NURBS or polygon mesh objects of these organs by our research group were volumetrically down‐scaled and fitted over the anatomically correct position. A model of the fetal skeleton generated from a former set of CT scans of an aborted 5-month fetus was imported into Rhinoceros. Since the fetal pelvis and mandible were segmented from MR images, the skeleton bones were rotated and rescaled to orient with those anatomy landmarks.

### Maternal breast

During the pregnancy, certain organs, such as the breasts, will have increased masses. The RPI-P series of pregnant hybrid phantom published the volume of Breasts at 9-month gestation [9]. This value was used in the current model as the reference volume. The breasts' tissue from ICRP female voxel phantom was replaced with a scaled model from Makehuman open source software to represent the reference volume.

### Maternal spine and soft tissues

The polygon mesh object of the spine segmented from MR images and exported from 3D-Doctor does not provide sufficient anatomical details. So, the Rhinoceros ‘flow along curve’ tool was used to align and orient the base phantom polygon mesh model of the spine guided by the location of the segmented spine (Fig. B1). This technique was used to modify the spinal cord and vessels.
